# Effects of in ovo feeding of chlorogenic acid on antioxidant capacity of postnatal broilers

**DOI:** 10.3389/fphys.2023.1091520

**Published:** 2023-01-16

**Authors:** Yali Pan, Hai Lin, Hongchao Jiao, Jingpeng Zhao, Xiaojuan Wang

**Affiliations:** ^1^ Shandong Provincial Key Laboratory of Animal Biotechnology and Disease Control and Prevention, College of Animal Science and Technology, Shandong Agricultural University, Tai’an, China; ^2^ Key Laboratory of Efficient Utilization of Non-grain Feed Resources (Co-construction by Ministry and Province), Ministry of Agriculture and Rural Affairs, Tai’an, China

**Keywords:** broiler, chlorogenic acid, in ovo feeding, heat stress, antioxidant, epigenetic mechanism

## Abstract

In this study, chlorogenic acid (CGA) was injected into the amniotic cavity of chicken embryos to study the effects of in ovo feeding of CGA on the antioxidant capacity of postnatal broilers. On the 17th day of embryonic age, a total of 300 healthy broiler fertile eggs with similar weights were randomly subjected to five groups as follows; in ovo injection with 0.5 ml CGA at 4 mg/egg (4CGA) or 7 mg/egg (7CGA) or 10 mg/egg (10CGA), or sham-injection with saline (positive control, PC) or no injection (negative control, NC). Each group had six replicates of ten embryos. Six healthy chicks with similar body weights hatched from each replicate were selected and reared until heat stress treatment (35°C ± 1°C, 8 h/d) at 28–42 days of age. The results showed that there was no significant difference in the hatching rate between the groups (*p* > 0.05). After heat stress treatment, 4CGA group showed an improved intestinal morphology which was demonstrated by a higher villus height in the duodenum and a higher villus height/crypt depth ratio in the jejunum, compared with the NC group (*p* < 0.05). The antioxidant capacity of chickens was improved by in ovo feeding of CGA since 4CGA decreased the plasma content of malondialdehyde (MDA) (*p* < 0.05), whereas, it increased the superoxide dismutase (SOD), glutathione peroxidase (GPX), and catalase (CAT) activities compared with NC group (*p* < 0.05). Also, the MDA content of the different injection groups had a quadratic effect, with the 4CGA group having the lowest MDA content (*P*_*quadratic*_ < 0.05). In the duodenum, 4CGA injection significantly increased the mRNA expressions of *nuclear factor erythroid 2-related factor 2 (Nrf2), heme oxygenase 1 (H O -1), glutathione synthetase (GSS),* and *SOD1* compared to the NC and PC groups (*p* < 0.05). The mRNA expressions of *glutathione reductase (GSR)* and *GPX7* were significantly increased in all CGA-treated groups compared with the PC group (*p* < 0.05), while the mRNA expression of *CAT* was significantly increased by 4CGA group than the NC group (*p* < 0.05). The mRNA expressions of epigenetic-related genes, *ten eleven translocation 1 and 2 (Tet1 and Tet2),* and *DNA-methyltransferase 3 alpha (DNMT3A)* in the duodenum of 4CGA injected group was significantly increased compared with the NC and PC groups (*p* < 0.05). The mRNA expressions of *Nrf2*, *SOD1*, and *Tet2* showed a significant quadratic effects with the 4CGA group having the highest expression (*P*_*quadratic*_ < 0.05). In conclusion, in ovo feeding of CGA alleviated heat stress-induced intestinal oxidative damage. Injection with CGA of 4 mg/egg is considered most effective due to its actions in improving intestinal antioxidant capacity, especially in the duodenum. The antioxidant effects of in ovo CGA on postnatal heat-stressed broilers may be related to its regulation of epigenetic mechanisms. Thus, this study provides technical knowledge to support the in ovo feeding of CGA to alleviate oxidative stress in postnatal heat-stressed broilers.

## Introduction

In recent years, the beneficial properties of natural plants and herbal substances have increasingly gained research interest. Among them, polyphenols have attracted much attention because of their antioxidant properties. Chlorogenic acid is one of the most common polyphenolic compounds found in the diet. It widely exists in various fruits and vegetables, such as coffee, apple, blueberry, eggplant, and chicory (Clifford et al., 2017; Yan et al., 2017). In addition, chlorogenic acid is also an effective component of many traditional Chinese medicines, such as *Eucommia cortex, Lonicera japonica, FlosChrysanthemi indici* and *Artemisia capillaris* Thunb (Wang et al., 2020a). In animals, chlorogenic acid can improve growth performance, immunity, and intestinal barrier by enhancing biological functions such as anti-inflammatory, antibacterial, antioxidant, and regulating lipid metabolism (Chen et al., 2018a; Chen et al., 2018b). Some studies have also shown that chlorogenic acid can inhibit bacteria *in vitro*, therefore chlorogenic acid may partially replace antibiotics and hormonal drugs (Santana-Gálvez et al., 2017). Also, chlorogenic acid plays a protective role by regulating oxidative stress-related pathways. In rats with cerebral ischemia-reperfusion injury, it was demonstrated that chlorogenic acid can activate the nuclear factor erythroid 2-related factor 2 (Nrf2) pathway, promote the expression of *Nrf2* and *heme oxygenase 1 (H O -1)*, improve the activity of superoxide dismutase (SOD) and reduce the malondialdehyde (MDA) content (Liu et al., 2020).

In ovo feeding refers to providing growth-promoting compounds or nutrients at the embryonic stage to initiate epigenetic changes and improve the health and growth of poultry during incubation (Uni et al., 2005; Jha et al., 2019a; Bednarczyk et al., 2021). The embryo begins to take in amniotic fluid on the 17th day of embryonic age which is suitable for in ovo feeding. The nutrients are injected into the amniotic cavity of the chicken embryo and then absorbed by the intestine through embryo swallowing (Zhao et al., 2018; Zhang et al., 2020a). Although the development of the intestine is a precise and orderly process, the function of the intestine can only begin to develop after the embryo swallows amniotic fluid in the late incubation stage (Wang et al., 2020b). Therefore, nutrients injected into the amniotic cavity in the later stage of incubation can be transported to the intestine to improve intestinal development (Willemsen et al., 2010). Studies have shown that chlorogenic acid can act on its own or through its metabolites. In rats and humans, caffeic acid in plasma appears rapidly after intake of chlorogenic acid, indicating that chlorogenic acid is hydrolyzed in the upper part of the gastrointestinal tract (Azuma et al., 2000; Nardini et al., 2002). Using an *in situ* intestinal perfusion model, it was confirmed that chlorogenic acid was effectively absorbed in the small intestine of rats, hydrolyzed in the mucosa, and recovered as free phenolic acid in the plasma (Lafay et al., 2006a). Chlorogenic acid can also be absorbed in the stomach in its intact form in rats, and it was also identified in rats’ plasma shortly after the oral administration of a honeysuckle extract (Meng et al., 2004; Lafay et al., 2006b).

With global climate change, environmental change has become a significant challenge to animal production. Ambient temperature is the most important environmental factor affecting animal production and health, especially in the summer period in most subtropical and tropical areas. Poultry industry is one of the industries most affected by the increase in ambient temperature (Goel et al., 2021). High ambient temperatures beyond the thermal neutral range are one of the most lethal stressors in poultry breeding (Niu et al., 2009). In addition, due to feather coverage and the absence of sweat glands, poultry has a poor ability to regulate body temperature under high temperature environment and are particularly sensitive to heat stress (Hu et al., 2019). It is reported that heat stress can occur in poultry of all ages and different breeds (Nawab et al., 2018). A comparison between animals of different ages revealed that older animals (42 days old) were more susceptible to heat stress than younger animals (21 days old) such that, 21-day-old broilers have a greater antioxidant capacity than 42-day-old broilers when exposed to heat stress (Del Vesco et al., 2017). Heat stress can lead to decreased food intake and intestinal dysfunction (Garriga et al., 2006; Song et al., 2013). Therefore, maintaining intestinal health is crucial to reducing heat stress injury. Oxidative stress refers to the overproduction of reactive oxygen species (ROS) and the imbalance of antioxidant defense (Lykkesfeldt and Svendsen, 2007). During heat stress, the body regulates more blood flow to the skin rather than internal organs in order to facilitate heat dissipation, resulting in gastrointestinal hypoxia, which leads to ROS overproduction and then intestinal damage (Liu et al., 2016; Gupta et al., 2017; McGarry et al., 2018). In addition, heat stress can induce lipid peroxidation (Huang et al., 2015). It upsets the balance between oxidation and antioxidant systems (Zhang et al., 2018a), and eventually leads to oxidative stress. Studies have shown that the dietary addition of antioxidants can reduce intestinal damage caused by heat stress in poultry (Cheng et al., 2019; Yang et al., 2021). *In vitro*, heat stress is also widely used as an inducer of oxidative stress (Li et al., 2019; Yu et al., 2019).

To alleviate the problem of heat stress, several studies have investigated the dietary supplementation of antioxidant substances to heat-stressed broilers. Based on the reported antioxidant properties of chlorogenic acid and the effects of in ovo feeding on intestinal development, it is considered that the in ovo feeding of chlorogenic acid may offer an effective and cost-effective approach to relieving heat stress in poultry. Therefore, in the present study, in ovo feeding technology was used to supplement chlorogenic acid to chicken embryos. Thereafter, the hatched chickens were subjected to heat stress-induced oxidative stress in order to explore the effects and mechanisms of embryonic chlorogenic acid on the antioxidant capacity of postnatal broilers.

## Materials and methods

### Animals and treatments

A total of 300 healthy broiler fertile eggs (Arbor Acres, *Gallus domesticus*) with similar body weight (average 59 g) were purchased from a local hatchery (Shandong Liuhe Breeding Co., Ltd, China) and were randomly divided into five groups, with six replicates in each group and 10 eggs in each replicate. All eggs were set in a single incubator, and six replicates (i.e. 6 trays) of each treatment group were randomly distributed on six layers of the incubator. Amniotic cavity injection was performed at 17 days of embryonic age with 0.5 ml chlorogenic acid (Sigma-Aldrich, Missouri, United States) of 4 mg/egg (4CGA) or 7 mg/egg (7CGA) or 10 mg/egg (10CGA) or sham-injected with saline (positive control group, PC), or non-injected group (negative control, NC). The dose of chlorogenic acid was calculated and determined according to a previous report by Chen (Chen et al., 2021a). The fertile eggs were incubated at 37°C under a relative humidity of 60%–70%.

Six healthy chicks with similar body weights from each replicate were selected after hatching and raised under intermittent heat stress treatment (35°C ± 1°C, 8 h/d, 60% relative humidity) during 28–42 days of age. Chicks were subjected to intermittent heat stress to better simulate the diurnal variation of ambient temperature during the summer season. The chicks were managed in environmentally controlled chambers and fed with commercial diets purchased from a local feed factory (Shandong Zhongcheng, China). Feed and water were provided *ad libitum*.

### Amniotic cavity injection

The in ovo injection of fertile eggs was performed in a sterile environment. A small electric drill was used to drill a hole with 1-mm diameter at the top of the egg air chamber, and a 1-ml syringe with a disposable sterile dental injection needle (0.5 × 38 TWLB L) was used to inject the solution into the amniotic cavity of the egg from the hole, with an injection depth of about 25 mm. The hole was sealed with paraffin after injection. The chlorogenic acid solution was prepared in normal saline and warmed to 37°C for injection.

### Samples collection

The hatching time, the number of chicks hatched, and the body weight of hatched chicks were recorded. After hatching, the weekly production performance of chickens during 1–27 d without heat stress and 28–42 d during heat stress were recorded. At the age of 27 d (before heat stress) and 42 d (after heat stress), eight healthy broilers with similar body weight were randomly selected from each treatment for weighing and sampling. After fasting for 6 h, blood was collected from the wing vein using 5-ml heparinized syringe. Plasma samples were obtained after centrifugation at 400 *g* for 10 min at 4°C and stored at -20°C for further analysis. Thereafter, the chicks were sacrificed by cervical dislocation and exsanguination. The liver, heart, duodenum, jejunum, ileum, breast muscle, and thigh muscle were isolated and weighed. The length of the duodenum, jejunum, and ileum was measured, and about 2 cm of the middle part of each small intestine segment was cut and fixed in 4% paraformaldehyde solution for histomorphological observation. Sections of the duodenum, jejunum, and ileum were sampled, immediately snap-frozen in liquid nitrogen, and stored at -80°C for further analysis.

### Plasma parameters analysis

Plasma activities of alanine aminotransferase (ALT), aspartate aminotransferase (AST), alkaline phosphatase (ALP), creatine kinase (CK), lactate dehydrogenase (LDH) and concentrations of total protein (TP) and albumin (ALB) were measured with commercial diagnostic kits (Sichuan Mike Biotechnology Engineering Co., Ltd, China). Plasma activities of SOD, catalase (CAT), glutathione peroxidase (GPX) and concentrations of MDA, total antioxidant capacity (T-AOC), and glutathione (GSH) were measured spectrophotometrically with commercial diagnostic kits (Nanjing Jiancheng Biological Engineering Research Institute, China).

### Intestinal histomorphological analysis

The intestinal histomorphology was observed with hematoxylin and eosin staining as described by Wang et al. (Huang et al., 2015). Briefly, the intestinal tissue was fixed conventionally in 4% paraformaldehyde, dehydrated, and embedded in paraffin. De-paraffinated sections with a thickness of 4 μm were stained with Harris hematoxylin and eosin (Sigma-Aldrich, Missouri, United States). Sections were examined under a Chongqing Mack Optoelectronic Instrument XDS-1B inverted microscope (Chongqing, China).

### Total RNA extraction and real-time PCR analysis

Total RNA was extracted from the intestine using commercial kit (Xinsaimei, Suzhou, China). Then the concentration of the RNA was measured by spectrophotometry (Eppendorf, Hamburg, Germany), and the RNA purity was verified by calculating the ratio between the absorbance values at 260 and 280 nm (A260/280 ≈ 1.75–2.01). Next, reverse transcription was performed using total RNA (1 μg) for cDNA synthesis with the kit (Cowin, Beijing, China). The cDNA was amplified in a 20-μL PCR reaction system according to the manufacturer’s instruction (Cowin, Beijing, China). Real-time PCR was performed at the ABI QuantStudio 5 PCR machine (Applied biosystems Inc., Carlsbad, CA). Real-time PCR was performed at 95°C for 30 s of predenaturation, followed by 40 cycles, with each cycle consisting of denaturation at 95°C for 5 s and annealing and extension at 60°C for 30 s. All samples were run in duplicate, and the specificity of the amplification product was verified by the standard curve and dissolution curve. Primers used in this study were designed using Primer 5.0 software and synthesized by Sangon Biotech (Shanghai, China, Table 1). Primers against GAPDH were used as internal controls, and mRNA expression was quantified according to the comparative CT method (2^-△△Ct^).

**TABLE 1:** Gene-specific primer of related genes.

Gene	Primer sequence	Accession NO.	Product size (bp)
*GAPDH*	F:ACATGGCATCCAAGGAGTGAG	NM_204305.2	144
	R:GGGGAGACAGAAGGGAACAGA		
*Nrf2*	F:GGGACGGTGACACAGGAACAAC	NM_205117.2	93
	R:TCCACAGCGGGAAATCAGAAAGATC		
*H O -1*	F:GGTCCCGAATGAATGCCCTTG	NM_205344.2	137
	R:ACCGTTCTCCTGGCTCTTGG		
*SOD1*	F:TTGTCTGATGGAGATCATGGCTTC	NM_205064.2	98
	R:TGCTTGCCTTCAGGATTAAAGTGAG		
*SOD2*	F:CAGATAGCAGCCTGTGCAAATCA	NM_204211.2	86
	R:GCATGTTCCCATACATCGATTCC		
*CAT*	F:TCTCATTCCAGTGGGCAAGATTGTC	NM_001031215.2	85
	R:GCTAGGGTCATACGCCATCTGTTC		
*GPX1*	F:TCACCATGTTCGAGAAGTGC	NM_001277853.3	124
	R:ATGTACTGCGGGTTGGTCAT		
*GPX7*	F:TTGCAATTACAGCACTCCTGCTC	NM_001163245.2	149
	R:TGCAACGTTGACAACTAACGACA		
*GSS*	F:GCTCAGTGCCAGTTCCAGTT	XM_040688004.1	115
	R:GGTCCCACAGTAAAGCCAAG		
*GSR*	F:CCAGAACACCACCAGAAAGG	XM_040671422.1	114
	R:TTACCAAAGAGCCGAAGTGC		
*DNMT1*	F:GGACGAGGATGAGGAGGTGGATG	NM_206952.1	95
	R:TGCGGCTCTTGTTCTGCTTCTTC		
*DNMT3A*	F:CCCTGGAGCACCCTTTGTTTATCG	NM_001024832.3	95
	R:CTGGTATCCGTCGTCATCGTATTGG		
*DNMT3B*	F:GACGGGAAGTTCTCTGAGGTTTCTG	NM_001396842.1	117
	R:ATGGTATATGGCTCGGCGGTAGG		
*Tet1*	F:GGGACAACCGACTGACTCTG	XM_040675306.1	195
	R:GAGATCCGCGTGGGATGATT		
*Tet2*	F:AGGCTATGGTGGTAGCCTCA	NM_001277794.3	164
	R:GAGCAGCGTGCTTGTGAAAA		
*Tet3*	F:GAAGGCGGTGAAGGAGGAGGAG	XM_040689495.1	91
	R:GTTCTCGTCCAGGAAGTTGTGCTC		

### Statistical analysis

Data analysis was performed by one-way ANOVA using SAS statistical software (SAS version 9.1; SAS Institute Inc., 2004). Differences between means were evaluated using Duncan’s Multiple Range test. All the values were expressed as means ± SE. Orthogonal polynomial contrasts were used for the trend test to examine the linear and quadratic effects of different chlorogenic acid injection concentrations. *p* < 0.05 means that the differences were significant, and 0.05 < *p* < 0.1 means that there was a tendency towards significance.

## Results

### Hatching performance

**FIGURE 1:**
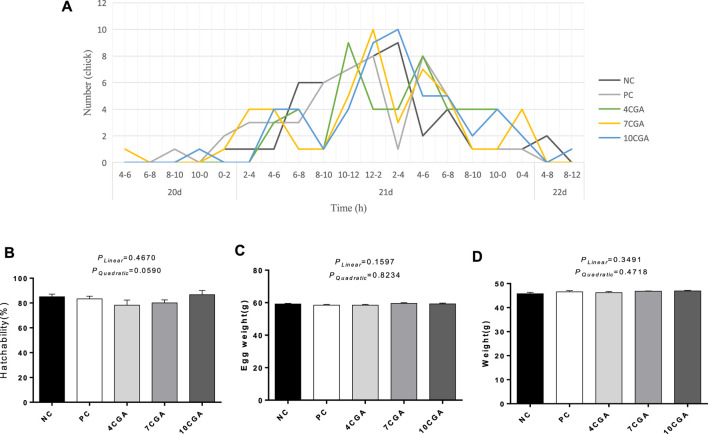
Effects of in ovo feeding of chlorogenic acid on hatching performance of broilers. **(A)** Hatching time; **(B)** Hatchability, *n* = 6; **(C)** Egg weight at hatching, *n* = 6; **(D)** Body weight of hatching broilers, *n* = 6.

The hatching peak time of the 4CGA group was 4 h earlier than that of NC group and 2 h earlier than that of PC group (Figure 1A). There was no significant differences in hatchability, egg weight at hatching, and body weight of hatching chicks among groups (*p* > 0.05, Figures 1B–D). There was no significant liner or quadratic effect (*P*_*linear*_ > 0.05; *P*_*quadratic*_ > 0.05).

### Production performance and mortality

There was no significant difference in the production performance between chlorogenic acid treatment and control (*p* > 0.05), but feed/gain at 1–27 d increased with the concentration of chlorogenic acid, with a linear effect (*P*_*linear*_ < 0.05). Although there was no significant difference between groups (*p* > 0.05), mortality was highest in the NC group (19.44%) and lowest in 4CGA group (8.33%). There was no significant quadratic effect (*P*_*quadratic*_ > 0.05) (Table 2).

**TABLE 2:** Effects of in ovo feeding of chlorogenic acid on production performance and mortality of broilers.

Treatment	NC	PC	4CGA	7CGA	10CGA	*p*-Value		
item						Treatments	Linear	Quadratic
1–27 days ofage(no heat stress)								
Feed intake, g/d	64.69 ± 1.55	68.10 ± 2.60	66.47 ± 0.63	67.18 ± 0.89	65.86 ± 1.57	0.6299	0.3830	0.9238
Body weightgain, g/d	35.66 ± 2.01	39.84 ± 1.41	38.76 ± 0.65	37.96 ± 1.19	36.69 ± 1.57	0.2891	0.0973	0.8541
Feed/gain g/g	1.84 ± 0.07	1.71 ± 0.01	1.72 ± 0.02	1.77 ± 0.03	1.80 ± 0.04	0.1821	0.0419	0.5823
28–42 days of age(heat stress)								
Feedintake, g/d	152.47 ± 4.87	146.23 ± 7.72	142.95 ± 8.14	150.10 ± 6.26	139.81 ± 7.30	0.7030	0.7314	0.6862
Body weightgain, g/d	70.10 ± 5.68	75.03 ± 3.86	69.18 ± 2.96	74.72 ± 4.83	71.50 ± 4.65	0.8407	0.7324	0.6823
Feed/gain, g/g	2.26 ± 0.24	1.95 ± 0.05	2.07 ± 0.11	2.03 ± 0.08	1.96 ± 0.05	0.4316	0.9216	0.1845
Mortality, %	19.44 ± 9.04	13.89 ± 6.69	8.33 ± 5.69	11.11 ± 5.56	16.67 ± 7.45	0.8116	0.7590	0.3939

Data were presented as mean ± SE (*n* = 6).

### Organ development

At 27 d when there was no heat stress, the heart proportion of broilers was significantly increased in 10CGA (0.48%) group than that in PC group (0.38%) (*p* < 0.05), the jejunum proportion had a linear effect (*P*_*linear*_ < 0.05), and no changes were observed for other organs (*p* > 0.05). In ovo feeding of chlorogenic acid had no significant effect on all organs weighed in the heat-stressed broilers at 42 d of age (*p* > 0.05). There was no significant quadratic effect (*P*_*quadratic*_ > 0.05) (Table 3).

**TABLE 3:** Effects of in ovo feeding of chlorogenic acid on organ proportion of broilers.

Treatment	NC	PC	4CGA	7CGA	10CGA	*p*-Value		
item (%)						Treatments	Linear	Quadratic
27 days of age (no heat stress)								
Liver	2.57 ± 0.08	2.48 ± 0.07	2.60 ± 0.04	2.50 ± 0.08	2.58 ± 0.15	0.9133	0.6497	0.7826
Heart	0.46 ± 0.03^a^ ^,^ ^b^	0.38 ± 0.05^b^	0.45 ± 0.02^a^ ^,^ ^b^	0.44 ± 0.02^a^ ^,^ ^b^	0.48 ± 0.02^a^	0.2372	0.0776	0.7460
Duodenum	0.73 ± 0.04	0.63 ± 0.02	0.67 ± 0.03	0.69 ± 0.02	0.66 ± 0.04	0.1992	0.1492	0.1685
Jejunum	1.23 ± 0.06	1.18 ± 0.06	1.21 ± 0.04	1.32 ± 0.02	1.27 ± 0.05	0.3135	0.0434	0.4188
Ileum	0.85 ± 0.04	0.74 ± 0.03	0.80 ± 0.03	0.81 ± 0.06	0.88 ± 0.05	0.2920	0.0659	0.8172
Breast muscle	14.67 ± 0.32	15.68 ± 0.60	15.60 ± 0.29	14.82 ± 0.52	15.27 ± 0.33	0.3684	0.3183	0.6735
Thigh muscle	14.23 ± 1.08	13.10 ± 0.29	13.08 ± 0.31	13.19 ± 0.23	13.17 ± 0.18	0.5021	0.8048	0.9756
42 days of age (heat stress)								
Liver	1.78 ± 0.10	1.77 ± 0.08	1.89 ± 0.08	1.78 ± 0.04	1.73 ± 0.06	0.6210	0.5666	0.1958
Heart	0.39 ± 0.02	0.39 ± 0.02	0.34 ± 0.02	0.39 ± 0.03	0.37 ± 0.02	0.4625	0.8212	0.3973
Duodenum	0.58 ± 0.03	0.57 ± 0.03	0.63 ± 0.05	0.58 ± 0.02	0.57 ± 0.03	0.7661	0.8115	0.3646
Jejunum	0.85 ± 0.05	0.89 ± 0.05	0.96 ± 0.05	0.84 ± 0.05	0.84 ± 0.03	0.3508	0.3097	0.2561
Ileum	0.58 ± 0.02	0.62 ± 0.02	0.62 ± 0.04	0.55 ± 0.02	0.60 ± 0.02	0.3245	0.2795	0.5382
Breast muscle	17.46 ± 0.66	15.64 ± 0.67	16.71 ± 0.70	16.55 ± 0.77	16.70 ± 0.51	0.4309	0.2196	0.3939
Thigh muscle	15.50 ± 0.25	15.62 ± 0.43	14.76 ± 0.46	15.94 ± 0.32	15.41 ± 0.47	0.3280	0.8583	0.5993

Data were presented as mean ± SE (*n* = 6).

^a^ Means within a row with different superscripts differ significantly (*p* < 0.05).

^b^ Means within a row with different superscripts differ significantly (*p* < 0.05).

For 27 d-broilers without heat stress, 4CGA group (277.68 mg/cm) had a higher jejunum index than NC group (243.76 mg/cm) (*p* < 0.05). Compared with NC (140.15 mg/cm) or PC group (140.34 mg/cm), the ileum index was significantly increased in the 4CGA group (163.09 mg/cm) (*p* < 0.05), while no changes were found in the intestinal organ index for 42 d-heat stressed broilers (*p* > 0.05). There was no significant liner or quadratic effect (*P*_*linear*_ > 0.05; *P*_*quadratic*_ > 0.05) (Table 4).

**TABLE 4:** Effects of in ovo feeding of chlorogenic acid on intestinal quality index of broilers.

Treat	NC	PC	4CGA	7CGA	10CGA	*p*-Value		
item (mg/cm)						Treatments	Linear	Quadratic
27 days of age(no heat stress)								
Duodenum	332.55 ± 12.32	338.92 ± 11.38	366.57 ± 15.15	368.81 ± 17.16	343.48 ± 15.32	0.2819	0.7189	0.0888
Jejunum	243.76 ± 2.46^b^	247.24 ± 13.56^ab^	277.68 ± 12.67^a^	271.99 ± 9.53^ab^	264.41 ± 9.39^ab^	0.0994	0.2796	0.0891
Ileum	140.15 ± 3.14^b^	140.34 ± 6.91^b^	163.09 ± 5.70^a^	144.49 ± 10.82^ab^	151.79 ± 4.66^ab^	0.1092	0.5218	0.2176
42 days of age(heat stress)								
Duodenum	451.16 ± 17.18	456.95 ± 17.01	484.97 ± 24.03	432.42 ± 15.80	476.46 ± 20.01	0.3276	0.9061	0.7859
Jejunum	332.11 ± 15.37	347.26 ± 18.88	331.05 ± 14.16	324.08 ± 26.75	348.84 ± 11.68	0.8258	0.9611	0.3115
Ileum	180.01 ± 4.52	174.85 ± 7.05	174.14 ± 11.92	165.99 ± 6.63	171.79 ± 4.01	0.7523	0.6308	0.7562

Data were presented as mean ± SE (*n* = 6).

^a^ Means within a row with different superscripts differ significantly (*p* < 0.05).

^b^ Means within a row with different superscripts differ significantly (*p* < 0.05).

### Plasma biochemical parameters

**FIGURE 2:**
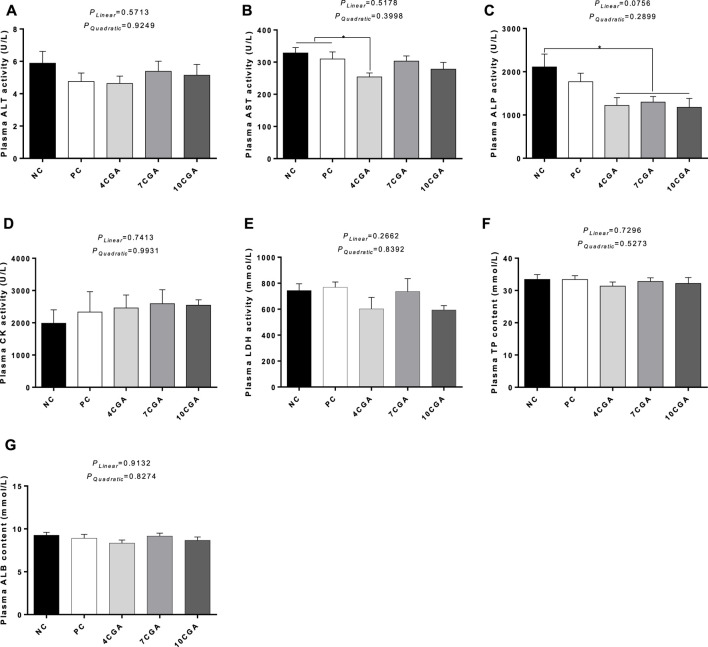
Effects of in ovo feeding of chlorogenic acid on plasma biochemical parameters of 42 d-heat stressed broilers. **(A)** ALT; **(B)** AST; **(C)** ALP; **(D)** CK; **(E)** LDH; **(F)** TP; **(G)** ALB. * indicates a significant difference between treatments (*p* < 0.05), *n* = 6.

For 42 d-heat stressed broilers, AST activity in the 4CGA group (253.88 U/L) decreased significantly compared with NC group (328.50 U/L) and PC group (309.75 U/L) (*p* < 0.05). The ALP content in all chlorogenic acid treated groups (average 1232.35 U/L) were significantly lower than that in NC group (2110.75 U/L) (*p* < 0.05). However, no changes were observed for other biochemical parameters (*p* > 0.05). There was no significant liner or quadratic effect (*P*_*linear*_ > 0.05; *P*_*quadratic*_ > 0.05) (Figure 2).

### Plasma oxidation and antioxidant parameters

**FIGURE 3:**
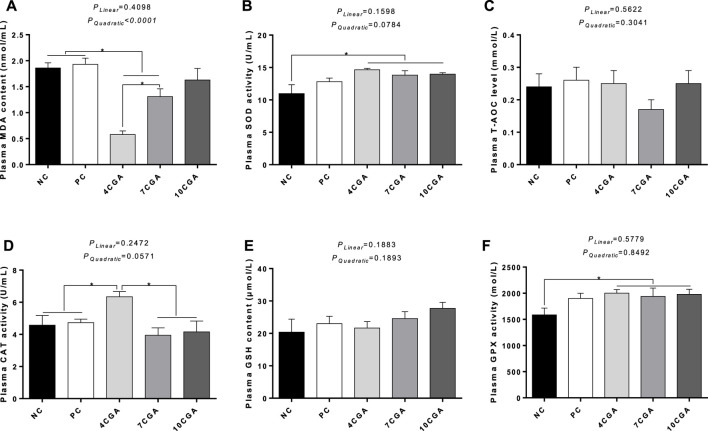
Effects of in ovo feeding of chlorogenic acid on plasma oxidation and antioxidant parameters of 42-heat stressed broilers. **(A)** MDA; **(B)** SOD; **(C)** T-AOC; **(D)** CAT; **(E)** GSH; **(F)** GPX. * indicates a significant difference between treatments (*p* < 0.05), *n* = 6.

For 42d-heat stressed broilers, the MDA content of 4CGA group (0.58 nmol/ml) and 7CGA group (1.31 nmol/ml) decreased significantly compared with NC group (1.86 nmol/ml) and PC group (1.93 nmol/ml) (*p* < 0.05). Also, the MDA content of 4CGA group (0.58 nmol/ml) was significantly lower than that of the 7CGA group (1.31 nmol/ml) (*p* < 0.05). The MDA content of the different injection groups had a quadratic effect, with the 4CGA group having the lowest MDA content (*P*_*quadratic*_ < 0.05). Compared with the NC group (SOD 10.97 U/mL; GPX 1585.95 mol/L), SOD and GPX activities in all chlorogenic acid-treated groups (average SOD 14.15 U/mL; GPX 1973.57 mol/L) were significantly higher (*p* < 0.05). The CAT activity in 4CGA group (6.34 U/mL) was significantly higher than that in control groups (average 4.66 U/mL) and other chlorogenic acid-treated groups (average 4.05 U/mL) (*p* < 0.05). However, there were no significant changes for T-AOC level and GSH content (*p* > 0.05). There was no significant liner effect (*P*_*linear*_ > 0.05) (Figure 3).

### Intestinal morphology

**FIGURE 4:**
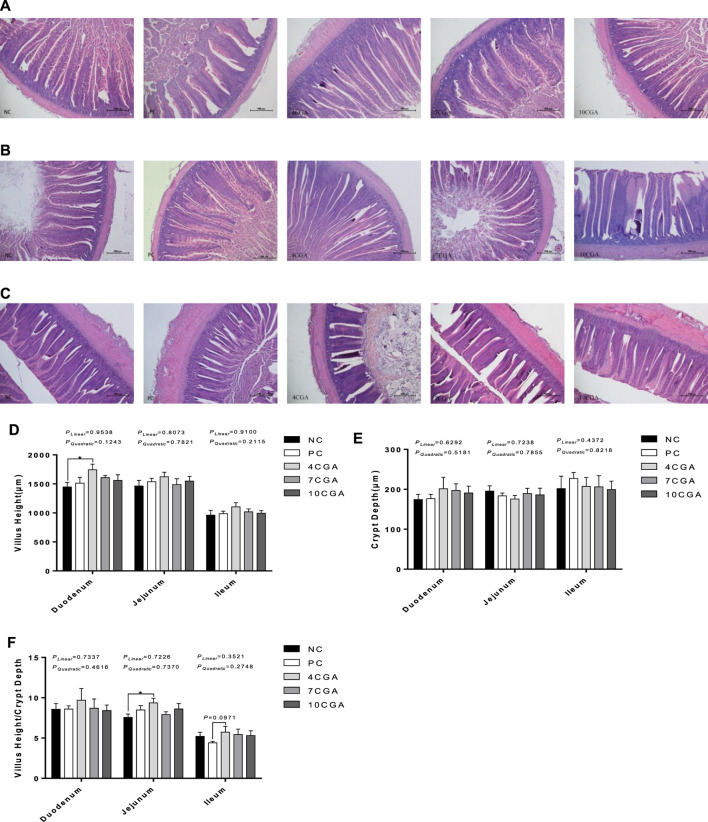
Effects of in ovo feeding of chlorogenic acid on intestinal morphology of 42 d-heat stressed broilers. **(A)** Duodenum; **(B)** Jejunum; **(C)** Ileum; **(D)** Villus Height; **(E)** Crypt Depth; **(F)** Villus Height/Crypt Depth. * indicates a significant difference between treatments (*p* < 0.05), *n* = 6.

For 42d-heat stressed broilers, the villus height of the duodenum in the 4CGA group (1743.57 μm) was significantly higher than that in the NC group (1447.81 μm) (*p* < 0.05, Figures 4A, D). In the jejunum, the ratio of villus height to crypt depth was significantly higher in the 4CGA group (9.36) than in the NC group (7.56) (*p* < 0.05, Figures 4B, F). In the ileum, the ratio of villus height to crypt depth in the 4CGA group (5.73) was higher than that in the PC group (4.40) (*p* = 0.0971, Figures 4C, F). There were no significant changes for crypt depth (*p* > 0.05, Figure 4E). There was no significant liner or quadratic effect (*P*_*linear*_ > 0.05; *P*_*quadratic*_ > 0.05).

### Intestinal mRNA expression

**FIGURE 5:**
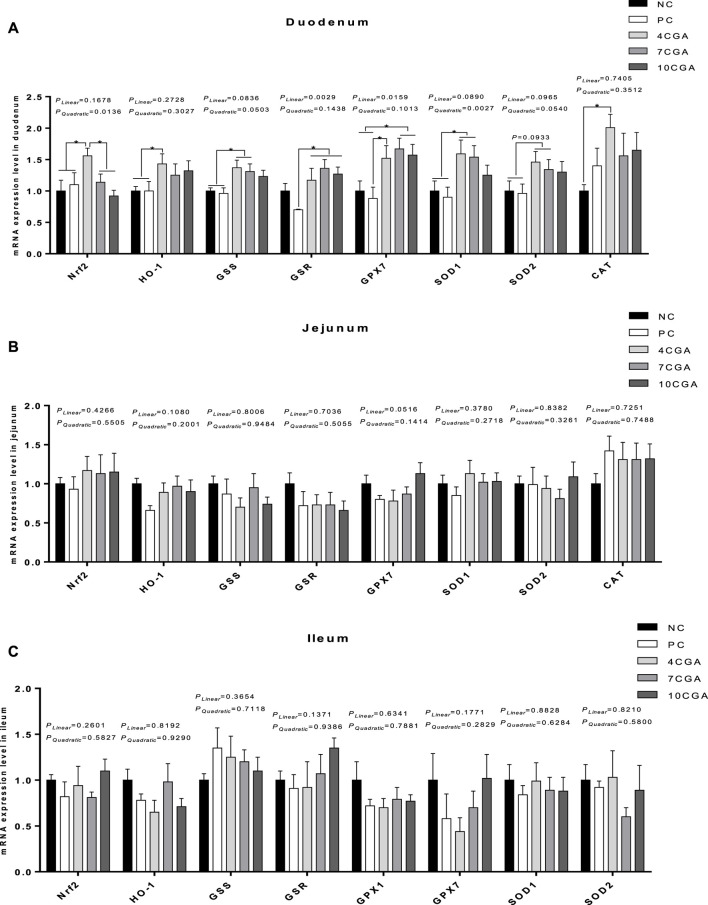
Effects of in ovo feeding of chlorogenic acid on mRNA expression of antioxidant-related genes of 42 d-heat stressed broilers. **(A)** Duodenum; **(B)** Jejunum; **(C)** Ileum. * indicates a significant difference between treatments (*p* < 0.05), *n* = 6.

In the duodenum of 42 d-heat stressed broilers (Figure 5A), the mRNA expressions of *Nrf2* and *H O -1* were significantly higher in the 4CGA group (1.56; 1.43) compared with NC (Yan et al., 2017Yan et al., 2017) and PC groups (1.1; 1) (*p* < 0.05). Also, the mRNA expression of *Nrf2* in the 4CGA group (1.56) was significantly higher than that in the 7CGA (1.14) and 10CGA (0.92) (*p* < 0.05) groups. The mRNA expressions of *glutathione synthetase (GSS)* and *SOD1* in the 4CGA group (1.37; 1.59) and 7CGA group (1.31; 1.54) were significantly higher than NC (Yan et al., 2017Yan et al., 2017) and PC groups (0.96; 0.9) (*p* < 0.05), while the mRNA expression of *SOD2* tended to increase (*p* = 0.0933). Compared with the PC group (0.7; 0.88), the mRNA expressions of *glutathione reductase (GSR)* and *GPX7* in all chlorogenic acid-treated groups (average 1.27; 1.59) were significantly higher (*p* < 0.05). More so, the mRNA expression of *GPX7* in 7CGA group (1.67) and 10CGA group (1.57) was significantly higher than that in NC group (Yan et al., 2017) (*p* < 0.05). The mRNA expressions of *Nrf2* and *SOD1* in different injection groups had a significant quadratic effect (*P*_*quadratic*_ < 0.05), and the 4CGA group was the highest. Also, the mRNA expressions of *GSR* and *GPX7* in different injection groups had a linear effect (*P*_*linear*_ < 0.05), and increased with the concentration of chlorogenic acid. The mRNA expression of *CAT* in the 4CGA group (2.01) was significantly higher than that in the NC group (Yan et al., 2017) (*p* < 0.05). However, there were no significant differences in mRNA expression of antioxidant-related genes between chlorogenic acid-treated groups and control groups (*p* > 0.05) in jejunum and ileum (Figures 5B,C).

**FIGURE 6:**
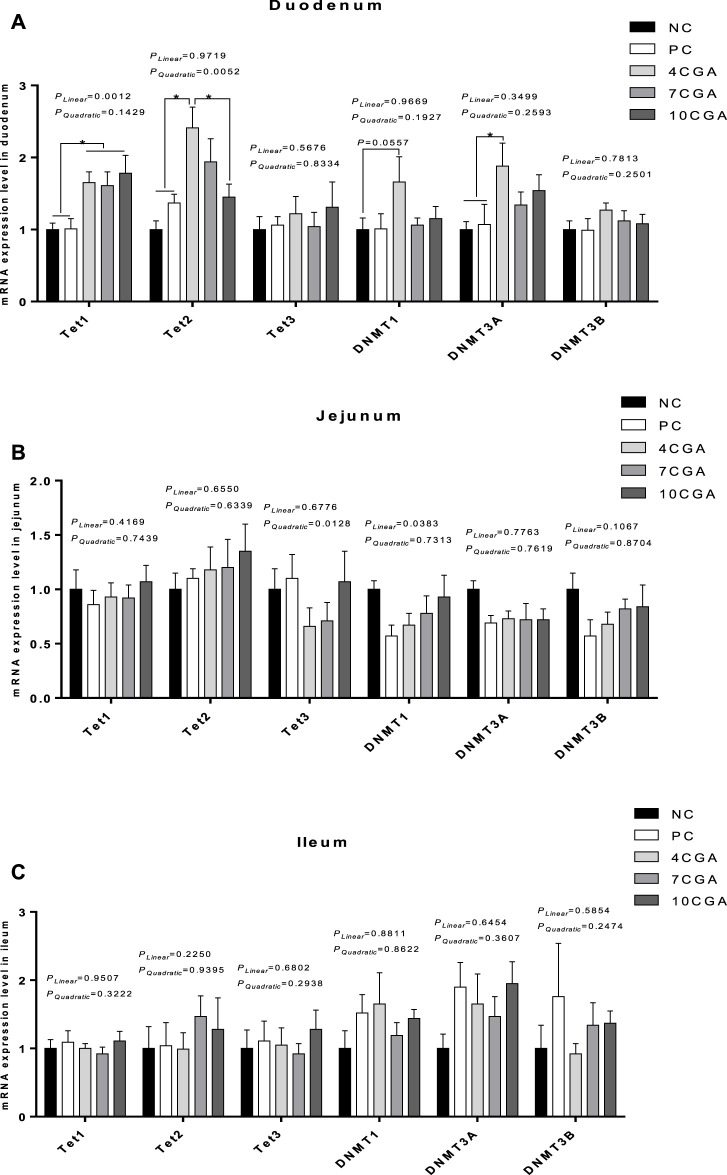
Effects of in ovo feeding of chlorogenic acid on mRNA expression of DNA methylation-related enzymes of 42d-heat stressed broilers. **(A)** Duodenum; **(B)** Jejunum; **(C)** Ileum. * indicates a significant difference between treatments (*p* < 0.05), *n* = 6.

Compared with NC (Yan et al., 2017) and PC group (1.01), the mRNA expression of *ten eleven translocation 1 (Tet1)* was significantly higher (*p* < 0.05) in all chlorogenic acid treated groups (average 1.68) in the duodenum of 42 d-heat stressed broilers (Figure 6A). The mRNA expression of *Tet2* and *DNA-methyltransferase three alpha (DNMT3A)* in the 4CGA group (2.41; 1.88) was also significantly higher than NC (Yan et al., 2017Yan et al., 2017) and PC group (1.37; 1.07) (*p* < 0.05), while the mRNA expression of *Tet2* in the 4CGA group (2.41) was significantly higher than that in 10CGA group (1.45) (*p* < 0.05). Compared with NC group (Yan et al., 2017), the mRNA expression of *DNMT1* in the 4CGA group (1.66) tended to increase (*p* = 0.0557). However, there were no significant differences in the mRNA expression of *Tet3* and *DNMT3B* between chlorogenic acid-treated groups and the control groups (*p* > 0.05); The mRNA expressions of *Tet2* in different injection groups had a significant quadratic effect (*P*_*quadratic*_ < 0.05), and the 4CGA group was the highest. Also, the mRNA expressions of *Tet1* in different injection groups had a linear effect (*P*_*linear*_ < 0.05), and increased with the concentration of chlorogenic acid. In jejunum and ileum (Figures 6B,C), there was no significant difference in the mRNA expression of DNA methylation-related enzymes between chlorogenic acid treated groups and control groups (*p* > 0.05); The jejunal mRNA expressions of *Tet3* in the different injection groups had a quadratic effect (*P*_*quadratic*_ < 0.05), such that the 4CGA group had the lowest mRNA expression. In addition, the jejunal mRNA expression of *DNMT1* had a linear effect (*P*_*linear*_ < 0.05) in different injection groups, thus, it was increased with increasing concentration of chlorogenic acid.

## Discussion

### Effects of in ovo feeding of chlorogenic acid on the growth, development and metabolism of postnatal broilers

Although several studies have discussed the importance of adding chlorogenic acid to broiler diets, the present experiment studied the effects of chlorogenic acid administration *via* in ovo feeding for the first time. The results showed that in ovo feeding of chlorogenic acid did not have an adverse effect on the hatching performance of broilers, thus ascertaining the reliability of the in ovo feeding technique.

Heat stress disturbs the body’s homeostasis, resulting in adverse effects on physiology, immunity, and intestinal morphology, with a consequent decrease in production and increased mortality of poultry (Xu et al., 2018). Chlorogenic acid has been previously used as an antioxidant due to its antioxidant properties. In the past few years, researchers have studied the dietary addition of chlorogenic acid, but its effects on growth performance were inconsistent. Chen et al. (Chen et al., 2021b) showed that adding chlorogenic acid to the diet for 2 weeks had no significant effect on the average daily gain and average daily feed intake of chicks with acute heat stress. However, Zhang et al. (2020b) found that after adding chlorogenic acid to the diet, the average daily gain was increased, and the average daily feed intake and feed/gain were significantly reduced for broilers infected by *Clostridium perfringens* during 1–21 days of age. Zhao et al. (2019) also showed that the dietary addition of *Eucommia ulmoides* leaf extract (which contained mainly chlorogenic acid) increased the average daily gain of broilers, and reduced the feed/gain, but had no significant effect on the average daily feed intake. In this study, there was no significant difference in production performance. During the whole feeding period, the mortality rate of 4CGA group was the lowest at 8.33%, while that of the control group was the highest at 19.44%. Chlorogenic acid had no effect on the growth performance of broilers, which may be due to the small population of experimental animals and the differences in experimental conditions, animal species, and mortality rate. The association between these factors needs to be further confirmed in future studies. Evidently, in ovo feeding of chlorogenic acid was beneficial to the development of the heart, jejunum, and ileum of postnatal broilers without heat stress.

The small intestine is the most important site for animals to digest and absorb nutrients (Lv et al., 2021). Intestinal mucosal function and health can be reflected by intestinal morphological indices, including villus height, crypt depth, and the ratio of villus height to crypt depth (Jha et al., 2019b). A higher villus facilitates nutrient absorption by the intestine, and a shallow crypt is beneficial to the maturation of intestinal epithelial cells (van der Hulst et al., 1998). An increased villus height/crypt depth ratio reflects an improvement in the intestinal mucosal structure and the enhancement of intestinal mucosal absorption function (Williams et al., 2015). Zhang et al. (Zhang et al., 2018b) showed that the dietary addition of chlorogenic acid had a positive effect on the intestinal morphology of weaned piglets, as demonstrated by the increased villus height and the villus height/crypt depth in duodenum and jejunum, whereas, the duodenal crypt depth was decreased. Similarly, Ruan et al. (2014) showed that the addition of chlorogenic acid can improve the intestinal morphology of the jejunum and ileum in lipopolysaccharide-treated rats. In the present study, 4CGA increased the villus height of the duodenum and the villus height/crypt depth of the jejunum and ileum compared to the control group after 2 weeks of heat stress. These results showed that in ovo feeding of chlorogenic acid had a positive effect on the intestinal morphology of postnatal broilers suffering from heat stress.

Blood biochemical indicators generally include blood glucose, liver function, and renal function indicators, which altogether reflect the health status of the body (Liao et al., 2007). Total protein mainly consists of albumin and globulin, and their ratio indicates protein metabolism and immune function (Stohl et al., 2019; Yoshino et al., 2019). Blood ALT, AST, and ALP are usually used to evaluate the degree of liver oxidative damage, and their decreased activity is indicative of an improved redox status (Dalia et al., 2017; Pandit et al., 2022). LDH and CK mainly exist in cardiomyocytes and can be released when cardiomyocytes are damaged. Therefore blood LDH and CK are important indicators to measure myocardial injury (Tang et al., 2018; Yin et al., 2020). The present results showed that, after 2 weeks of heat stress, the blood activity of AST and ALP were reduced in CGA-treated groups, especially in the 4CGA group. This suggests that in ovo feeding of chlorogenic acid can alleviate the oxidative damage of postnatal heat stress in the liver.

### Effects of in ovo feeding of chlorogenic acid on the antioxidant capacity of postnatal broilers

Heat stress upset the balance between oxidation and antioxidant systems thereby inducing lipid peroxidation, and ROS overproduction. As such, heat stress is widely used to establish both *in vivo* and *in vitro* models of oxidative stress (Huang et al., 2015; Liu et al., 2016; Gupta et al., 2017; Zhang et al., 2018a; McGarry et al., 2018; Li et al., 2019; Yu et al., 2019), as presented in this study.

Antioxidant enzymes, especially SOD and CAT can inhibit the accumulation of reactive oxygen species and protect cells from oxidative stress. T-AOC reflects the overall antioxidant capacity of the organism (Chen et al., 2012). GSH is an important antioxidant that scavenges hydrogen peroxide and ROS (Wang et al., 2015), while GPX is a powerful free radical scavenger and an important enzyme in the antioxidant defense system (Zhang et al., 2018b). MDA is the oxidation product of lipid peroxide, and the MDA content can directly reflect the level of lipid peroxide and the degree of lipid peroxidation caused by free radicals (Lv et al., 2021). As a polyphenol, chlorogenic acid acts as an effective neutralizer of ROS by scavenging free radicals (Yao et al., 2019). Studies have shown that chlorogenic acid can increase the serum level of T-AOC, promote the activities of SOD and GPX, and inhibit MDA content, effectively alleviating oxidative stress caused by *Clostridium perfringens* infection (Zhang et al., 2020b). Consistently, Zhou et al. (2016) found that chlorogenic acid can increase the activities of GPX and SOD and the level of T-AOC in H_2_O_2_-induced intestinal mitochondrial injury in rats. The molecular mechanism of antioxidant activity of chlorogenic acid may depend on its specialized chemical structure. Chlorogenic acid can provide hydrogen atoms to reduce free radicals, inhibit oxidative reactions, and then rapidly stabilize resonance after oxidation (Liang and Kitts, 2015). In this study, after 2 weeks of heat stress, the plasma oxidation product MDA was significantly decreased and showed a significant quadratic effect, with the 4CGA group having the lowest MDA levels. The activities of antioxidant enzymes including SOD, GPX and CAT were significantly increased by the 4CGA group, indicating that in ovo feeding of chlorogenic acid would play an antioxidant role *via* increasing the activities of antioxidant enzymes.

We further explored the underlying mechanism of chlorogenic acid’s antioxidant effect. Nrf2 is an important transcription factor that regulates the gene expression of antioxidant enzymes (Kim et al., 2010). The Nrf2 pathway exerts an antioxidant effect by up-regulating a series of endogenous protective genes (Wu et al., 2017). HO-1 is one of the key cytoprotective enzymes in cell defense. It is a key protein and plays an important role in cell adaptation to oxidative stress caused by a variety of pathological events (Hwang and Jeong, 2010; Ibáñez et al., 2014). Many studies have confirmed that the up-regulation of HO-1 contributes to cellular defense against oxidative damage (Lee et al., 2012; Xu et al., 2015; Han et al., 2017). Liu et al. (2020) found that chlorogenic acid promoted the protein expressions of Nrf2 and HO-1, increased SOD activity, and reduced MDA concentration by activating the Nrf2 pathway in rats with cerebral ischemia-reperfusion injury. The present study showed that, after 2 weeks of heat stress, the mRNA expressions of *Nrf2* and antioxidant enzyme *H O -1, GSS, GSR, GPX7, SOD1,* and *CAT* in the duodenum of 4CGA group were significantly increased, while the mRNA expression of *SOD2* also tended to increase. In addition, the mRNA expressions of *GSR* and *GPX7* in different injection groups showed a linear effect. The mRNA expressions of *Nrf2* and *SOD1* had a significant quadratic effects, and the 4CGA group was the highest. These results showed that chlorogenic acid activated the Nrf2 pathway and promoted the mRNA expressions of *Nrf2* and *H O -1*, thus improving the expression of their downstream antioxidant enzymes. Altogether, the results indicate that in ovo feeding of chlorogenic acid improved the antioxidant capacity of postnatal broilers subjected to heat stress by regulating the Nrf2 pathway.

### Effects of in ovo feeding of chlorogenic acid on epigenetics of broilers

Epigenetics refers to heritable changes in gene expression that are not caused by changes in DNA sequences (Holliday, 1987). It mainly includes DNA methylation, RNA interference, and histone modification (Egger et al., 2004), among which DNA methylation is the most famous (Yin et al., 2013). DNA methylation is catalyzed by DNA methyltransferases (DNMTs) at cytosine in the CpG sequence. During DNA replication, a methyl is added to the cytosine in the complementary DNA strand of methylated cytosine by DNMT1, and a methyl is added to the unmodified cytosine in the CpG sequence by DNMT3A and DNMT3B (Li et al., 1992; Okano et al., 1999). In addition, methylated cytosine is oxidized by Tet1, Tet2, and Tet3 of the Tet family, especially in the early stage of mouse embryonic development, and transformed into 5-hydroxymethylcytosine (Tahiliani et al., 2009; Inoue and Zhang, 2011). Genomic analysis showed that the chicken genome was rich in methylated cytosine, and contained homologous genes of mouse DNMT1, 3A, 3B; and Tet1, 2 and 3 (Li et al., 2011; Tada et al., 2021). In this study, the mRNA expressions of *Tet1*, *Tet2,* and *DNMT3A* in the duodenum of the 4CGA group were significantly increased, while the mRNA expression of *DNMT1* in the 4CGA group also tended to increase. The mRNA expressions of *Tet2* in the different injection groups showed a quadratic effect with the 4CGA group having the highest expression. This indicates that the effects of in ovo feeding of chlorogenic acid may be related to the epigenetics of broilers.

## Conclusion

This study reveals that in ovo feeding of chlorogenic acid can alleviate heat stress-induced oxidative damage in the intestinal tissue by promoting the antioxidant defense capacity of broilers. Importantly, chlorogenic acid injection with 4 mg/egg was found to be the most effective. The antioxidant effects of in ovo feeding of chlorogenic acid in postnatal heat-stressed broilers may be related to epigenetics, however, the underlying mechanism needs to be further explored.

## Data Availability

The original contributions presented in the study are included in the article/supplementary material, further inquiries can be directed to the corresponding author.
